# A Manual of Percussion and Auscultation

**Published:** 1876-10

**Authors:** 


*A Manual of Percussion and Auscultation.* By Austin Flint, M. D. Philadelphia: Henry C. Lea, 1876. Buffalo: T. Butler & Son.

In this little work Dr. Flint has given to the profession the substance of the lessons which he has given to his private classes for many years upon physical diagnosis. The reputation of the author, as a master of the subject, will make the work at once popular with students and physicians. The subject is treated in a clear, familiar manner, and is at the same time concise in all its details, no one seeking for information on these topics can leave this work, after diligent study, without having gained a large fund of knowledge.

